# Targeted cellular ionic calcium chelation by oxalates: Implications for the treatment of tumor cells

**DOI:** 10.1186/1475-2867-12-51

**Published:** 2012-12-08

**Authors:** Abraham Embi, Benjamin J Scherlag, Peter J Embi, Manuel Menes, Sunny S Po

**Affiliations:** 1 Miami, Florida, USA; 2 Health Sciences Center, Heart Rhythm Research Institute, Department of Medicine, University of Oklahoma, Oklahoma City, Oklahoma, USA; 3 Departments of Biomedical Informatics & Internal Medicine, The Ohio State University, Columbus, Ohio, USA; 4 Pathopinion, Delray Beach, Florida, USA

**Keywords:** Oxalates, Calcium chelation, Calcium sequestration, Apoptosis, Cancer cells, Metastatic melanoma, Astrocytes, Blood–brain barrier

## Abstract

**Background:**

In malignant melanoma, it has been published that up to 40% of cancer patients will suffer from brain metastasis. The prognosis for these patients is poor, with a life expectancy of 4 to 6 months. Calcium exchange is involved in numerous cell functions. Recently, three types of cellular calcium sequestration have been reported in the medical literature. The first describes a transgenic mouse model in which an increase of aberrant calcium channels triggers hypertrophy and apoptosis. The second provides a protective mechanism whereby astrocytes in the brain inhibit apoptosis of tumor cells by moving ionic calcium out of the tumor cells thru gap junctions. The third is via calcium chelation, which causes cell apoptosis by converting ionic calcium into a calcium salt. This process has been shown to operate in atrial myocardial cells, thus not allowing the intracellular calcium stores to flow through the myocytes intercalated discs. Ideally chemotherapeutic agents would be those that initiate apoptosis in tumor cells.

**Presentation of the Hypothesis:**

We hypothesize that the recent reported intracellular calcium sequestration by oxalate chelation, due to its chemical process of converting ionic calcium into a calcium salt, may inhibit the protective effect of astrocytes on brain tumor metastasized melanoma cells by not allowing free calcium to leave the metastatic cells, simultaneously apoptosis of tumor and some healthy adjacent cells could occur. This hypothesis could be extended to include other cancerous tumors such as skin cancers amongst others.

**Testing the hypothesis:**

Using the experimental model showing the protective mechanism of co-cultured reactive astrocytes and tumor cells treated with oxalates could be used to test this hypothesis in vitro. The calcium specific von Kossa technique could be used to confirm the presence of chelated intracellular calcium architecture of the metastatic cells (which is a sign of apoptosis), and extracellular calcium chelation stores of the Astrocytes (which has been shown to slow neural conduction).

**Implications of the Hypothesis:**

The life expectancy in patients with metastasized malignant melanoma brain tumors could be significantly prolonged if the chemotherapeutic issue of brain metastasis is overcome. Other cancerous tumors can also be treated by this Targeted Chelation Approach. Ionic calcium sequestration using naturally occurring calcium chelators, viz., oxalates, could accomplish this desired outcome.

## Background

### Ionic calcium increase induces apoptosis in a transgenic model

At the turn of the century (2001), it was reported that hypertrophy and apoptosis had occurred in cardiac cells of a Ca++ dependent transgenic model of cardiac hypertrophy. This model manifested an increased number of L-type calcium channels that provided an increased ingress of calcium thereby initiating hypertrophy and apoptosis [1].

### Protective effects of ionic calcium sequestration by physiologic processes: astrocytes

Cancer chemotherapeutic agents can promote apoptosis in tumors, attenuating their growth and metastasis. However, in some cases these chemotherapeutic effects can be attenuated by physiologic instances of calcium sequestration. Temporal analyses of melanoma cells incubated with chemotherapeutic agents have revealed an increase in cytoplasmic calcium followed by the appearance of fragmented DNA, one of the hallmarks of apoptosis [2]. However, activated astrocytes in the brain seem to protect metastatic melanoma tumor cells from apoptosis by a form of ionic calcium sequestration. This has been shown *in vitro* to be dependent on physical contact at gap junctions between cultured rat astrocytes and metastasized melanoma tumor cells that act by reducing the intracellular ionic calcium concentration in the tumor cell rather than by converting ionic calcium to calcium carbonate [3].

### Effects of ionic calcium sequestration by chelation

Ionic calcium can also be sequestered via chelation to achieve certain cellular effects. In recent experiments, we studied the effect of various millimolar per liter (mM/L) concentrations of oxalates, namely oxalic acid (OA) and ammonium oxalate (NH4Ox), on the intrinsic cardiac autonomic nervous system of dogs. An incidental finding was the infiltration of oxalates into the adjacent atrial myocardium resulting in various degrees of intracellular and extracellular calcium chelation. This revealed that in the heart, particularly in the intra-neural connections and at the axon-myocytes junction, calcium sequestration by oxalate chelation converts the intracellular myocytes’ ionic calcium stores as well as the extracellular ionic calcium of the neural network into calcium carbonate. This conversion inhibits neural conduction and neurotransmitter release, and this might yield a positive outcome in a patho-physiological situation with hyperactive neurons in the ganglionated plexi, which leads to atrial fibrillation [4].

**Figure 1:**
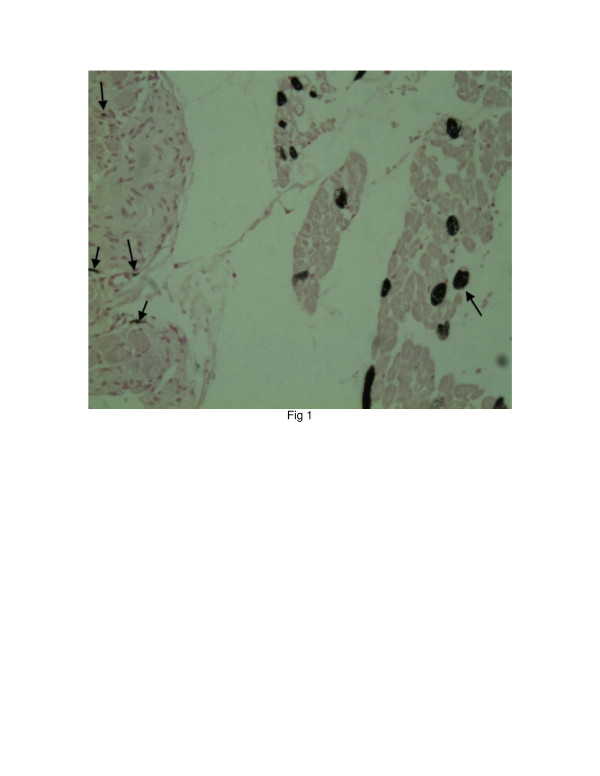
**Calcium chelated cross section of atrial myocardium adjacent to ganglionated plexus.** Stained via von Kossa technique. Slide clearly denotes intracellular stained calcium in myocytes and extracellular chelation of ganglion and nerves. After injection of 100 mM/L of NH4Ox. Arrows (right side of image) showing chelated cytoplasmic ionic calcium stores of myocytes. Arrows (left side of image) denote extracellular calcium stores chelation of ganglion and nerves. Fan Y, Scherlag BJ, Embi A et al. Neural Effects of Oxalic Acid for Atrial Fibrillation Therapy. 33^r*d*^*Annual Scientific Session Heart Rhythm.* 2012; 9:S258.

**Figure 2:**
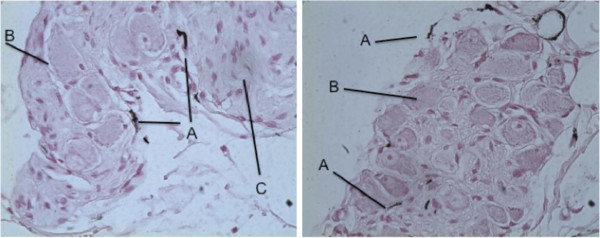
**Nerve and parasympathetic ganglion of intrinsic cardiac nervous system X 40 Magnification.** Showing heterogeneous extracellular calcium chelation. Post injection of 100 mM/L of Oxalic Acid.Black lines denote: **A** extracellular calcium chelation along edges of neurons and in ganglion/nerve bundle junction. **B**) Ganglion cell. **C**) Nerve bundle. Fan Y, Scherlag BJ, Embi A et al. Neural Effects of Oxalic Acid for Atrial Fibrillation Therapy. 33^r*d*^*Annual Scientific Session Heart Rhythm.* 2012; 9:S258.

Results from another, ongoing study suggests that chelation occurs irrespective of the pH of the chelators, ranging from OA (pH1.6) to NH4Ox (pH 6.7) (Embi A, Scherlag BJ unpublished observations). The control and post-injected samples were treated with a specific stain for calcium via the Von Kossa technique [5]. All control samples were negative for calcium chelation, on the other hand all other microinjected samples were positive for varying degrees of chelation (Figures 1 and 2).

## Presentation of the hypothesis

The recent reported intracellular calcium sequestration by oxalate chelation, due to its chemical process of converting ionic calcium into a calcium salt, may inhibit the protective effect of astrocytes on brain tumor metastasized melanoma cells by not allowing free calcium to leave the metastatic cells. This could be accomplished by simultaneously apoptosis of malignant cells in the area targeted. Adjacent non-malignant cells physiological function could be affected by the process of chelation. This hypothesis could be extended to include other cancerous tumors such as skin cancers amongst others.

## Testing the hypothesis

Using the experimental model showing the protective mechanism of co-cultured reactive astrocytes and tumor cells treated with oxalates could be used to test this hypothesis in vitro. The calcium specific von Kossa technique could be used to confirm the presence of chelated intracellular calcium architecture of the metastatic cells (which is a sign of apoptosis), and extracellular calcium chelation stores of the Astrocytes (which has been shown to slow neural conduction).

Oxalate calcium chelation could inhibit the astrocytes from sequestering ionic calcium from the cytoplasm of tumor cells. As per our findings on the oxalate infiltration and calcium sequestration in atrial myocytes, there should be a significant attenuation in the reactive astrocyte’s protective ability for the melanoma tumor cells. Flow measurements *in vitro* of the cytosolic calcium in oxalate treated metastatic melanoma cells could support this theory. *In vivo* experimental approach is the use of iron-clad nanoparticles with an oxalate payload. Since nanoparticles are reported to cross the blood–brain barrier [6], calcium chelators in this case can be selectively deployed by an outside force, such as magnetism aimed at the tumor area.

## Implications of the hypothesis

In malignant melanoma, it has been published that up to 40% of cancer patients will suffer from brain metastasis [7]. The prognosis for these patients is poor, with a life expectancy of 4 to 6 months [8]. The life expectancy could be significantly prolonged if the chemotherapeutic issue of brain metastasis is overcome. Furthermore, just as radiation therapy with the “gamma-knife” technique enables targeted therapeutic effects not possible with less-targeted radiation approaches, so too would targeted intracellular chelation via tailored superparamagnetic ferrous covered nanoparticles (MNP_S_) crossing the blood brain barrier which can be electively guided and deployed by internal or external force or stimuli [9].

We are not advocating a systemic administration of calcium chelators, with the consequent deleterious effects [10], instead a novel use of *in situ* microinjection or perhaps a targeted chelation approach delivered Targeted delivery of endogenous substances such as OA (pH 1.6) and NH4Ox (pH 6.7) should be considered for inclusion in research protocols of some existing cancer treatments.

We conclude that the existing published medical literature on the extensive correlation of calcium and cancer treatment supports and justifies further research using this novel approach.

## Abbreviations

Calcium chelation: Chemical process of substances binding to Ca^++^ forming a calcium salt such as Calcium Carbonate (CaCO_3_); mM/L: Denotes concentration of millimoles per liter; MNP_S_: Superparamagnetic nanoparticles made with Fe_3_O_4_ (core), thermoresponsive polymeric hydrogel (shell); NH4Ox: Ammonium Oxalate; OA: Oxalic Acid.

## Competing interests

The authors declare that they have no competing interests.

## Authors’ contributions

AE Hypothesis and preliminary calcium chelation work, drafting of manuscript, final decision. BJS Research chelation design, original chelation experiments and invaluable support. PJE Substantial contribution, guidance, drafting and essential focusing of manuscript. MM Pathology Histology staining and pathologist interpretation. SSP Laboratory Director, funding and guidance as to selecting Ammonium Oxalate as preferred endogenous substance. All authors read and approved the final manuscript.

## Authors’ information

Abraham Embi- BS- MBA/HA. Associate Consultant, Miami, Florida USA. Benjamin J Scherlag PH D – Professor of Medicine, University of Oklahoma, Heart Rhythm Institute, Oklahoma City, Oklahoma, USA. Peter J. Embi MD FACR Associate Professor of Medicine, Rheumatology. Associate Director Informatics. The Ohio State University, Columbus, Ohio USA. Manuel Menes MD Pathology Consultant- Path Opinion, Delray Beach, Florida, USA. Sunny S Po MD PH D Professor of Medicine, Director Heart Rhythm Institute, University of Oklahoma, Oklahoma City, Oklahoma, USA.
